# Radiotherapy and surgery remain effective treatment options for retroperitoneal MPNST: a retrospective study based on SEER database

**DOI:** 10.3389/fsurg.2024.1339170

**Published:** 2024-05-30

**Authors:** Zhe Xi, Zhuang Aobo, Xi Li, Wang Yue, Guangting Yan, Zhenhang Lin, Geng Zhang, Xiaogang Xia, Lanlan Lian, Wengang Li

**Affiliations:** ^1^ School of Medicine, Cancer Research Center, Xiang'an Hospital of Xiamen University, Xiamen University, Xiamen, Fujian, China; ^2^ College of Arts and Science, Boston University, Boston, MA, United States; ^3^ Department of Hepatobiliary and Pancreatic & Organ Transplantation Surgery, School of Medicine, Xiang'an Hospital of Xiamen University, Xiamen University, Xiamen, Fujian, China; ^4^ Department of Laboratory Medicine, Xiang'an Hospital of Xiamen University, Xiamen University, Xiamen, Fujian, China

**Keywords:** malignant peripheral nerve sheath tumours, retroperitoneal tumors, SEER database, retrospective study, radiotherapy

## Abstract

**Introduction:**

The proportion of retroperitoneal malignant peripheral nerve sheath tumours (RMPNST) in retroperitoneal tumors is less than 5%, but the mortality rate is very high. However, there is no relevant research focused on RMPNST only.

**Methods:**

We retrospectively analyzed data from the SEER database of patients with primary RMPNST from 2000 to 2019, by leveraging the advantages of the Seer database, we can explore the prognosis of such rare diseases. Kaplan-Meier method was used to construct the survival curve, and cox regression model was used to analyze the factors affecting the prognosis of patients. In addition, a model was developed to distinguish high-risk and low-risk patients.

**Results:**

This study included a total of 52 patients, with a median survival time of 39 months (95% CI 12.740–65.260) and a 5-year survival rate of 44.2% (95% CI 0.299–0.565). Radiotherapy (*p* = 0.004, OR: 1.475, 95% CI 0.718–3.033), metastasis disease (*p* = 0.002, OR: 5.596, 95% CI 2.449–47.079) and surgery (*p* = 0.003, OR: 5.003, 95% CI 0.011–0.409) were associated with overall survival (OS). The 5-year distant metastasis rate was 36% (95% CI 0.221–0.499). We used the above risk factors to separate patients into high and low groups and evaluate the results through the receiver operating characteristic (ROC) curve. This model is beneficial for guiding the selection of treatment strategies.

**Conclusion:**

The majority of RMPNST patients have a good prognosis after surgery, and the establishment of high-low group is helpful for clinical decision-making.

## Background

Soft tissue sarcoma (STS) is a rare type of cancer that originates from the mesenchymal tissue and comprises over 50 different histological subtypes (1). Retroperitoneal soft tissue sarcomas (RPS) are rare tumours which account for approximately 12%–15% of all STSs with a mean incidence of 2.7 per million (2, 3). Malignant peripheral nerve sheath tumors (MPNSTs) make up approximately 4% of all STSs and are known for their propensity for recurrence and poor prognosis (3, 4). The incidence rate of RMPNST in the population is about 0.000001%.

MPNSTs arise from benign peripheral nerve plexiform neurofibromas that originate in the embryonic neural crest cell lineage (5). The 8%–13% of individuals with NF1 mutations that develop MPNST constitute nearly 50% of all MPNST cases (6, 7), and MPNST is the leading cause of death in NF1 (8). Of the remaining cases, 45% of MPNSTs occur sporadically with unidentified genetic anomalies, and the rest are associated with radiotherapy (6). MPNST can occur at any age and there is no difference in occurrence between genders. However, it tends to appear earlier in life compared to other sarcomas with genomic complexity, which are typically more common in people over the age of sixty (9).

MPNSTs mainly occur in the head and neck region or upper extremities, with only 1% of cases located in the retroperitoneal region (10). The prognosis of MPNST is generally poor, with high rates of relapse following multimodality therapy in early disease, low response rates to cytotoxic chemotherapy in advanced disease, and propensity for rapid disease progression and high mortality (11). To date, surgery is the only proven therapy increasing survival in localized MPNSTs (12–14), while complete surgical resection is the primary treatment for MPNST it is often hindered by the large size of tumors, their proximity to complex nerve networks, and a low rate of negative resection margins (6, 11, 15). They are not sensitive to chemotherapy and radiotherapy and tend to recur locally (10). To date, no clinical trial with targeted agents for MPNST has demonstrated substantial tumor shrinkage or prolongation in progression-free survival (16).

Since there is currently no cohort study focused on RMPNST, this article will explore the factors that affect the prognosis of RMPNST by summarizing the treatment of 52 patients with RMPNST in the SEER database, and thus provide a relatively reliable treatment recommendation.

## Methods

We queried the SEER Research Plus Data, 17 Registries, Nov 2021 Sub (2000–2019), for patients diagnosed with Malignant Peripheral Nerve Sheath Tumor (variable: AYA site recode 2020 Revision coded as “MPNST”) occurring in the retroperitoneum (variable: site recode ICD-O-3/WHO 2008 coded as “Retroperitoneum”).

Clinical and demographic characteristics were evaluated as follow: gender, age, race, marital status at diagnosis, median household income inflation adjusted to 2019, tumor burden (median and range in mm), cause of death, survival months (median and range), primary tumor is MPNST or not, number of cancer cases, distant metastasis of tumor, sequence of chemotherapy and surgery, and sequence of surgery and radiotherapy. Race was classified into three groups: Black, White, and Other Marital status at diagnosis was categorized into three groups: Married (including common law), Single (never married), and Other. Household income, adjusted for inflation to 2019 is divided into the following three groups: $35,000–$54,999, $55,000–$74,999, $75,000+ FNCLCC (French Federation of Cancer Centers Sarcoma Group Grading System) grade was categorized into four groups: Grade 1, Grade 2, Grade 3, and Unknown. Cause of death was classified into three groups: Survived, Died due to MPNST, and Other causes of death. In terms of the relationship between chemotherapy and surgery, it can be divided into four groups: preoperative chemotherapy group, postoperative chemotherapy group, simultaneous preoperative and postoperative chemotherapy group, and surgery only or chemotherapy only group. Surgery and radiotherapy are also divided into four groups: preoperative radiotherapy group, postoperative radiotherapy group, intraoperative radiotherapy (IORT) group, and radiotherapy only or surgery only group. Continuous variables included age, survival time, median household income inflation adjusted to 2019 and tumor size.

## Statistical methods

Overall Survival (OS) rates were calculated using Kaplan-Meier and compared by log-rank tests. We use the Kaplan-Meier method in SPSS software to plot survival curves for each single factor. Univariate cox proportional hazards analysis was performed to evaluate the impact of various clinicopathological factors on prognosis, and variables with a *p*-value <0.1 or clinically relevant to patient prognosis were further included in the multivariate cox model (17). All tests were two-tailed, and *p* < 0.05 was considered statistically significance. All data were analyzed using SPSS 26.0 (SPSS Inc. Chicago, IL, USA).

## Results

### Patient and tumor characteristics

**Figure 1:**
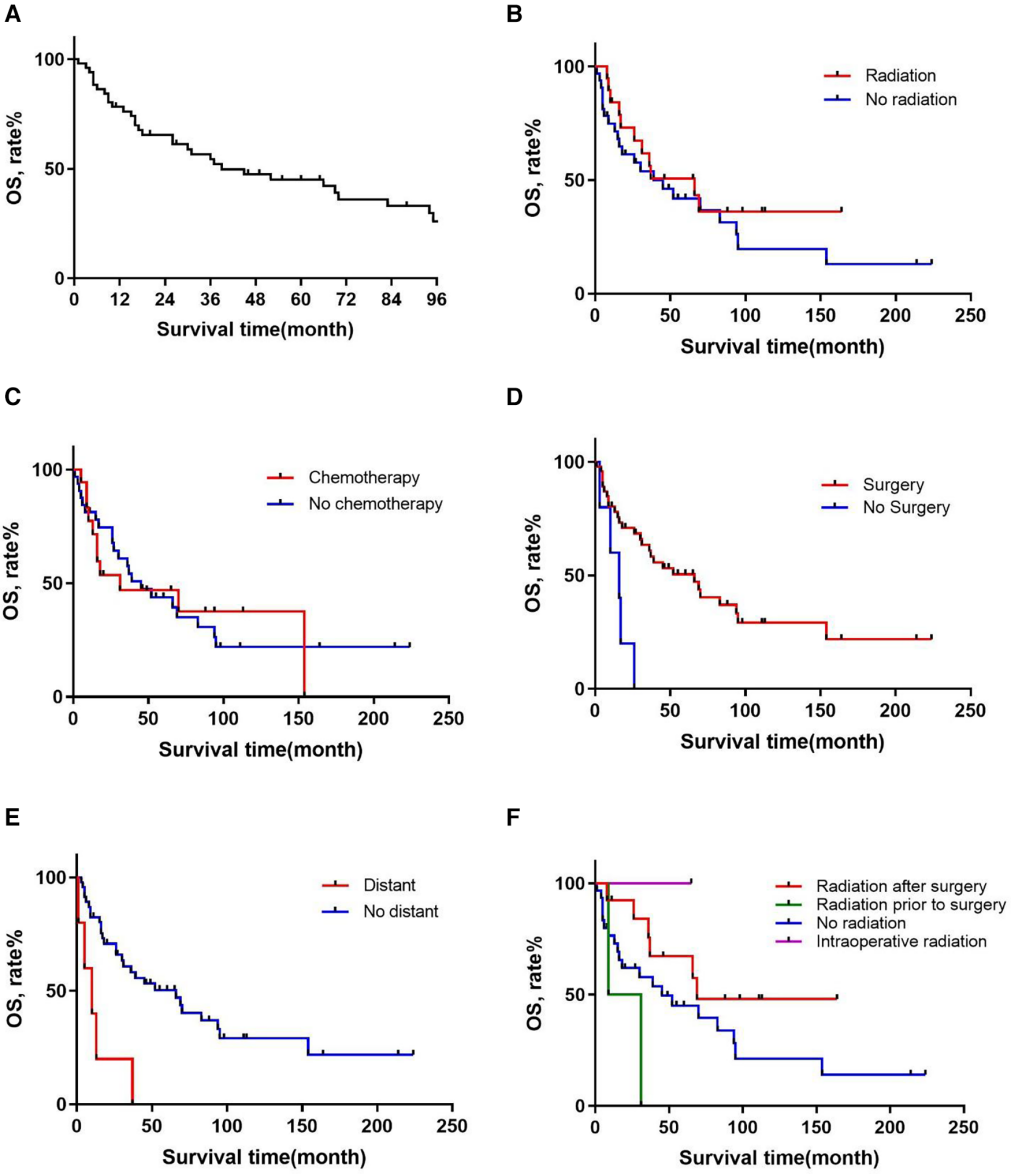
Overall survival in patients with primary RMPNST by (**A**) all patients, (**B**) radiation, (**C**) chemotherapy, (**D**) surgery, (**E**) distant metastasis, (**F**) sequence of radiation and surgery.

A total of 52 patients met the inclusion criteria, with a median followup time of 98 (range 0–224) (95% CI 73.298–122.702) months. 34 (65%) patients were dead at the last followup. The median OS time was 39 months (95% CI 12.740–65.260). The OS rates were 76.7% at 1 year, 60% at 2 years, and 44.2% at 5 years (Figure 1).

Patient characteristics are presented in Table 1. Of these patients, 27 (52%) were male and 25 (48%) were female with a median age of 46 (range, 12–79) years. Regarding the FNCLCC grading of MPNST, 1 (2%) patient was grade 1, 7 (13%) were grade 2, 26 (50%) were grade 3, and 18 (35%) had unknown grading. As for treatment with radiotherapy and chemotherapy, 19 (37%) patients underwent radiotherapy and 18 (35%) received chemotherapy. The median tumor burden was 130 (range, 47–250) mm. In terms of survival, there were 18 (35%) cases were still alive, 16 (31%) cases died of MPNST, and 18 (35%) cases died of other reasons. There were 6 (12%) cases with distant metastasis. Regarding the sequence of chemotherapy and surgery, 1 (2%) patient received chemotherapy before surgery, 7 (13%) patients received surgery before chemotherapy, 2 (4%) patients received chemotherapy both before and after surgery, and 42 (81%) patients only underwent surgery or chemotherapy. As for the sequence of surgery and radiotherapy, 13 (25%) patients received radiotherapy after surgery, 2 (4%) patients received radiotherapy before surgery, only 1 (2%) patient received radiotherapy during surgery, and 36 (69%) patients did not undergo surgery or radiotherapy.

**Table 1:** Patient and tumor characteristics of 52 primary RMPNSTs patients.

Characteristics	Number (*N* = 52)	% of total all
Gender		
Male	27	52
Female	25	48
Age, years median (range)	46	
Race		
White	40	77
Black	5	10
Other	7	13
Marital status at diagnosis		
Married (including common law)	20	38
Single (never married)	27	52
Other	5	10
Household income inflation adj to 2019		
$35,000–$54,999	13	25
$55,000–$74,999	22	42
$75,000+	17	33
FNCLCC		
Grade 1	1	2
Grade 2	7	13
Grade 3	26	50
Unknown	18	35
Radiation		
Yes	19	37
No	33	63
Radiation prior to surgery	2	4
Radiation after surgery	13	25
Intraoperative radiation	1	2
No radiation or No surgery	36	69
Chemotherapy		
Yes	18	35
No	34	65
Chemotherapy before surgery	1	2
Chemotherapy after surgery	7	13
Chemotherapy both before and after surgery	2	4
No surgery or no chemotherapy	42	81
Tumor burden, mm median (range)	130	
Cause of death		
Alive	18	35
MPNST	16	31
Other cause of death	18	35
Survival months median (range)	31	
Primary tumor is MPNST		
Yes	42	81
No	10	19
Surgery		
Yes	46	88
No	6	12
Distant metastasis of tumor		
Yes	6	12
No	46	88

### Univariable survival analyses

Table 2 presents the results of the univariate cox proportional hazards analysis of important prognostic factors affecting OS. There was a difference in survival between patients who underwent surgery (*p* = 0.001) and those who did not, with a median survival time of 36.5 months vs. 13 months (95% CI 1.900–13.174) (Figure 1). There was also a difference in survival between patients with and without tumor metastasis (*p* = 0.001) detected, with a median survival time of 7.5 (95% CI 0.877–1.007) and 39 months (95% CI 18.586–59.414), respectively (Figure 1). Subsequently, we used the Kaplan-Meier method to estimate the survival curve of each potential prognostic factor. We found that chemotherapy (*p* = 0.905) was ineffective for the prognosis of patients with RMPNST. As for radiotherapy, although the *p*-value of radiotherapy did not reach statistical difference (*p* = 0.290), there was a trend of survival rate difference between the two groups observed from the survival curve (Figure 1), which should be due to insufficient sample size.

**Table 2:** Univariable and multivariable analyses to determine independent predictors of OS of primary RMPNSTs.

Variables	Univariate analysis	*P*-value	Multivariate analysis	*P*-value
	Hazard ratio (95% CI)		Hazard ratio (95% CI)	
Gender female vs. male	0.879 (0.447–1.731)	0.710		
Age (continuous)	0.997 (0.979–1.016)	0.781		
Race		0.836		
White vs. Black	0.780 (0.183–3.325)	0.737		
White vs. other	1.253 (0.479–3.276)	0.646		
Marital status at diagnosis		0.931		
Married vs. single	0.876 (0.427–1.797)	0.719		
Other vs. single	0.880 (0.257–3.012)	0.838		
Household income inflation adj to 2019		0.771		
$35,000–$54,999 vs. $75,000+	0.713 (0.284–1.790)	0.471		
$55,000–$74,999 vs. $75,000+	0.885 (0.415–1.885)	0.751		
Radiation yes vs. no	1.475 (0.718–3.033)	0.290	0.183 (0.057–0.587)	0.004
Sequence of radiation and surgery		0.221		
Radiation after surgery vs. no radiation	2.665 (0.596–11.909)	0.199		
Radiation prior to surgery vs. no radiation	0.500 (0.200–1.251)	0.139		
Intraoperative radiation vs. no radiation	0.000 (0.000-)	0.982		
Chemotherapy yes vs. no	1.045 (0.508–2.149)	0.905	0.635 (0.211–1.910)	0.419
Sequence of chemotherapy and surgery		0.436		
Chemotherapy before surgery vs. no chemotherapy	0.392 (0.091–1.687)	0.208		
Chemotherapy after surgery vs. no chemotherapy	1.824 (0.233–14.298)	0.567		
Chemotherapy both before and after surgery vs. no chemotherapy	0.000 (0.000-)	0.987		
Blank vs. no chemotherapy	1.754 (0.697–4.415)	0.233		
Distant site yes vs. no	5.596 (2.179–14.372)	0.001	10.737 (2.449–47.079)	0.002
Total number of in malignant tumors for patient		0.490		
1 vs. 2	1.445 (0.675–3.093)	0.344		
1 vs. 3	0.719 (0.213–2.426)	0.595		
Surgery yes vs. no	5.003 (1.900–13.174)	0.001	0.066 (0.011–0.409)	0.003
FNCLCC		0.814		0.227
Grade 2 vs. grade 1	0.436 (0.048–3.961)	0.461	0.068 (0.005–0.903)	0.042
Grade 3 vs. grade 1	0.688 (0.090–5.258)	0.719	0.171 (0.017–1.707)	0.133
Unknown vs. grade 1	0.583 (0.074–4.572)	0.607	0.122 (0.010–1.448)	0.096
Tumor burden (continuous)	1.002 (0.993–1.012)	0.652	1.004 (0.994–1.015)	0.437
Number of cancer cases		0.685		
2 (MPNST was the first discovered) vs. 1	1.047 (0.391–2.800)	0.928		
2 (MPNST was the second discovered) vs. 1	1.562 (0.649–3.760)	0.320		
3 vs. 1	0.543 (0.073–4.062)	0.552		

### Multivariable survival analyses

In addition, we conducted a multivariate cox proportional hazards analysis, mainly including factors reported in previous literature and associated with prognosis of retroperitoneal sarcoma, such as surgery, metastasis, FNCLCC grade, radiation therapy, chemotherapy, and tumor size. The results were consistent with the univariate analysis and survival curves, indicating that surgery (*p* = 0.001), metastasis (*p* = 0.002), and radiotherapy (*p* = 0.004) had a significant impact on patient prognosis, while chemotherapy (*p* = 0.419), FNCLCC grading (*p* = 0.227), and tumor size (*p* = 0.437) had little effect on the prognosis of patients with RMPNST. Interestingly, in the FNCLCC grading of RMPNST, the comparison between Grade 1 and Grade 2 patients in the multivariable survival analyses was significant (*p* = 0.042).

Furthermore, for patients who received both surgery and radiation therapy, we compared the survival curves for those who received preoperative radiation, postoperative radiation, intraoperative radiation, and no radiation therapy (Figure 1). Thirteen patients received postoperative radiation therapy and 33 patients did not receive radiation therapy. The median survival times for the postoperative radiation group and the no radiation group were 66 and 26 months (HR: 2.221, 95% CI 0.964–5.121, *p* = 0.061), respectively. The survival curves also showed a trend towards a difference in survival between the two groups. Compared to patients who did not receive radiation therapy, those who received postoperative radiation had a significantly better prognosis.

**Figure 2:**
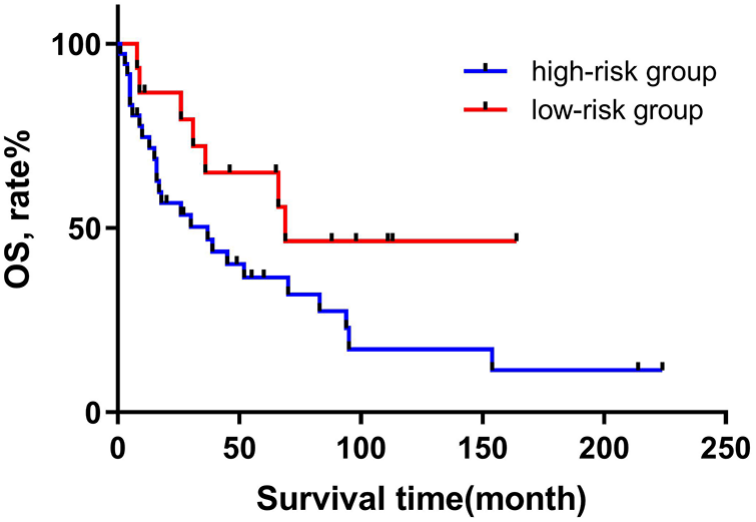
Overall survival in patients with primary RMPNST by high-low risk group classification.

**Figure 3:**
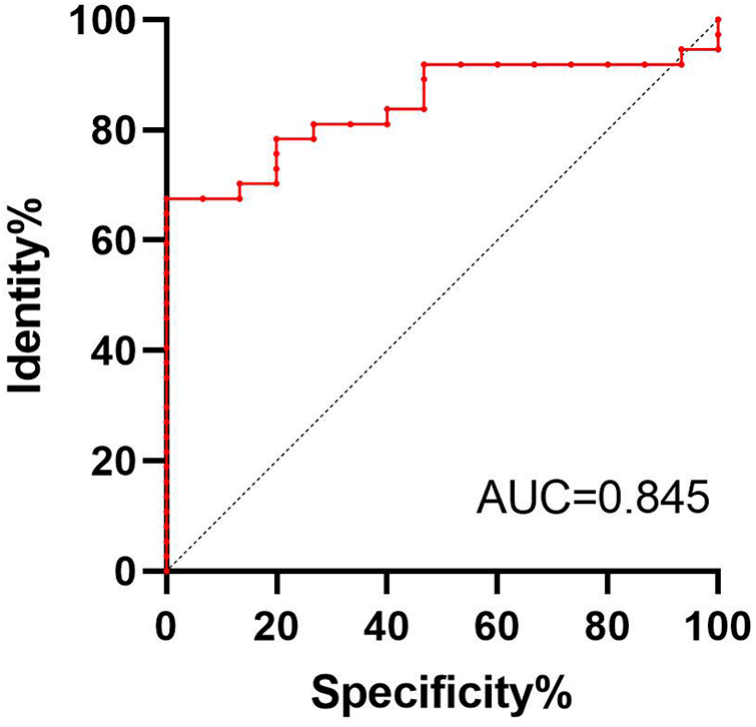
ROC curve analysis of high and low risk groups.

Based on the above research, we consider surgery, tumor metastasis, and radiotherapy as important factors that affect the prognosis of patients with RMPNST. Therefore, we divided the 52 patients in this study into a high-risk group and a low-risk group. The high-risk group was defined as patients who met at least one of the following criteria: 1. did not receive surgical treatment; 2. experienced tumor metastasis; 3. did not receive radiotherapy. Patients who did not meet any of the above criteria were included in the low-risk group. 37 patients were divided into the high-risk group. We then used the Kaplan-Meier method to generate survival curves for the high-risk and low-risk groups (Figure 2). The median survival times for the high-risk and low-risk groups were 67.5 and 21 months (*p* = 0.139, 95% CI 0.184–0.744, 95% CI 0.224–0.656), respectively. Nonetheless, based on the survival curves, we can conclude that there is a trend towards a difference in survival between the high-risk and low-risk groups, and the prognosis for the low-risk group is significantly better than that for the high-risk group. And the discriminability of the model was evaluated using the C index and the receiver operating characteristic (ROC) curve (Figure 3), AUC = 0.845. Therefore, we believe that this grouping method is meaningful.

## Discussion

MPNST pose a significant clinical challenge due to their aggressive nature as soft-tissue sarcomas (18). The incidence rate of MPNST is approximately one in a million (19). The WHO previously used terms such as “malignant Schwannoma”, “neurofibrosarcoma”, and “malignant neurofibroma” to indicate the origin and malignant behavior of the tumor. In order to avoid confusion, the WHO changed these terms to “malignant peripheral nerve sheath tumor” in 2002. In 2013, MPNST was classified as a type of soft tissue tumor (20). Patients with MPNST occurring in the retroperitoneum account for approximately 5.5% of the total (12). In the retroperitoneum, MPNST may arise from the vertebral nerve roots or from the nerves of either the sacral or lumbar plexus (21, 22). To date, there has been no systematic investigation in medical literature of MPNSTs that arise in the retroperitoneal region. Therefore, this study represents the first cohort study on RMPNST. Our findings demonstrate the positive impact of surgery and radiotherapy on the treatment of RMPNST, and a risk stratification model have been established based on the results. In addition, RMPNST may differ from the extremity and needs to be treated separately during treatment.

### Compared to MPNSTs in other parts of the body

This study presents the first cohort analysis of patients with MPNSTs occurring in the retroperitoneum. Based on a dataset of 52 RMPNST cases from the SEER database, we conducted a comprehensive analysis of individual characteristics and surgical status as risk factors affecting OS. In comparison to Enrico Martin's report of 594 non-RMPNST patients (12), our study found a higher proportion of RMPNST patients receiving radiation therapy (37% vs. 45%), but a lower proportion receiving chemotherapy (35% vs. 6.1%). Additionally, we found that the median survival time for RMPNST patients was shorter (39 vs. 72 months) despite comparable gender distribution and surgical removal rates. Compared with 374 head and neck MPNST patients reported by Armin Arshi (23), this study found that patients with RMPNST had a younger median age (46 years vs. 52 years), a larger tumor burden (130 mm vs. 39 mm), a lower proportion of patients receiving radiation therapy (37% vs. 42.5%), a lower 5-year survival rate (44.2% vs. 51%), and a shorter median survival time (39 months vs. 75.6 months), while there was no significant difference in gender and surgical resection rate. These results indicate that patients with RMPNST have a worse prognosis when compared to those with MPNST occurring in other areas. This may be attributed to the larger tumor burden and lower proportion of patients receiving adjuvant therapy in RMPNST cases.

### Chemotherapy

The role of chemotherapy in the treatment of RPS is unkonwn (24). In a multicenter randomized controlled trial conducted by the European Organization for Research and Treatment of Cancer (EORTC) Soft Tissue and Bone Sarcoma Group and the Sarcoma Disease Site Committee of the National Cancer Institute of Canada (NCIC) on 351 patients who underwent surgical resection of soft tissue tumors, patients who received chemotherapy had a 10% higher 5-year progression-free survival rate compared to those who did not receive chemotherapy. However, there was no significant difference in OS rate and local recurrence rate between patients who received or not. Moreover, patients who received chemotherapy had a significantly higher incidence of toxic side effects than those who did not receive chemotherapy (25). However, according to the study by Pervaiz N, chemotherapy can significantly improve both disease-free survival and OS in patients with locally resectable soft tissue sarcoma. Moreover, most of the adverse effects of chemotherapy are mild and can be effectively managed and treated (26). Another study, conducted by the Soft Tissue and Bone Sarcoma Group of the European Organization for Research and Treatment of Cancer, included over 300 patients. They found that MPNST patients had a poorer response to first-line chemotherapy (with doxorubicin and ifosfamide) and had a shorter survival time compared to other subtypes of soft tissue sarcoma. Among MPNST patients, those who received first-line chemotherapy had a slightly longer survival time, but the difference was not significant. In addition, the study found that the treatment outcome of MPNST was closely related to the tumor differentiation degree, that is, the lower the differentiation degree, the poorer the treatment effect and the shorter the survival time (27). There are findings that utilizing cytotoxic agents such as doxorubicin and ifosfamide for neoadjuvant therapy can render some localized, initially inoperable primary MPNSTs operable, thereby enhancing disease-free survival (28). Additionally, in a study examining patients with stage III/IV MPNSTs, a regimen combining ifosfamide with doxorubicin or etoposide managed to stabilize the disease in most cases (29). However, the overall response rates to chemotherapy in patients with advanced MPNST remain modest, generally between 20% and 30% (18, 29, 30).

In terms of targeted therapy, unfortunately, the clinical efficacy of non-cytotoxic targeted treatments for MPNST remains limited, with less than 25% of patients achieving stable disease and a median progression-free survival of less than two months (31). Moreover, inhibitors of angiogenesis are considered among the most promising therapeutic approaches for MPNST, underscoring that combination drug regimens are more effective than single-agent therapies. Specifically, while monotherapy with tyrosine kinase inhibitors (TKIs) targeting angiogenesis-related receptors (such as cabozantinib, imatinib, sunitinib) significantly reduces tumor growth and vascularization in various MPNST mouse models, the most effective treatment tested was the combination of sorafenib with doxorubicin or an mTOR inhibitor (18, 31).

In our study, there were a total of 18 patients (35%) who received chemotherapy. We considered whether chemotherapy was a factor affecting prognosis and performed a univariate analysis and generated a survival curve (*p* = 0.905). After generating the survival curve, it became clear that chemotherapy was not significantly associated with patient prognosis. In terms of 5-year survival, 11 patients (11/34) who did not receive chemotherapy survived for more than 5 years, while 6 patients (6/18) who received chemotherapy survived for more than 5 years, with similar proportions. The use of chemotherapy drugs, either preoperatively or postoperatively, had limited effects on patient survival time. In summary, the commonly used chemotherapy drugs in clinical practice do not significantly improve the OS rate and 5-year survival rate of patients with RMPNST, and the drug reactions caused by chemotherapy drugs can reduce patients' immune response, cause gastrointestinal discomfort, and decrease their quality of life. Therefore, considering the negative results of chemotherapy and the side effects of chemotherapy in this study, the use of chemotherapy should be cautious, especially for patients with early resectable disease.

### Radiation therapy

This study also investigated the impact of radiotherapy on the prognosis of patients with RMPNST. In terms of statistical analysis, we used univariate cox proportional hazards analysis to evaluate radiotherapy as a factor influencing prognosis, and obtained the hezard ratio is 1.475 (95% CI 0.718–3.033, *p*-value = 0.290). Meanwhile, we used the Kaplan-Meier method to plot survival curves and observed a trend of survival difference between the radiotherapy group and the non-radiotherapy group. Subsequently, we performed a multivariate Cox proportional hazards analysis on radiotherapy, yielding a *p*-value of 0.004, indicating statistical significance. Taking these findings into consideration, we firmly believe that radiotherapy is an important factor influencing the prognosis of RMPNST patients.

After confirming the therapeutic effect of radiotherapy, we further investigated the impact of postoperative radiotherapy and surgical order on patient prognosis. Using univariate cox proportional hazards analysis and the Kaplan-Meier method to plot survival curves, we found that patients who received postoperative radiotherapy (HR: 2.665, 95% CI 0.596–11.909, *p* = 0.199) had a trend towards survival difference compared to those who did not receive radiotherapy, although the *p*-value did not reach statistical significance. Therefore, we believe that in patients with RMPNST, surgical treatment followed by postoperative radiotherapy can significantly improve patient prognosis and quality of life. According to the results of our study, postoperative radiotherapy may be an effective way, which needs to be confirmed by further prospective clinical trials with large sample sizes.

In the research conducted by Stucky CC and Zou C, they found that the local recurrence rate of MPNST was approximately 22%–26% (13, 32). However, in the study by Anghileri M, the local recurrence rate of MPNST exceeded 50% (33). This may be related to the highly invasive nature of MPNST itself and differences in its malignant degree. Meanwhile, a study by Alia Mowery of over 1,000 cases of MPNST found that the metastasis rate of MPNST was approximately 3.7% (34), indicating that the recurrence pattern of MPNST is mainly local recurrence, with relatively few distant metastases. According to the research by Sylvie Bonvalot, neoadjuvant radiation therapy has limited effectiveness in RPS and should only be considered for well-differentiated liposarcoma (WDLPS), especially for the WDLPS subtype with local recurrence (35). In the previous discussion, we also found that MPNST was consistent with the general recurrence pattern of RPS, so adding radiotherapy may have a positive effect on the treatment of MPNST originating from the retroperitoneum. In our research, we have also arrived at similar conclusions.

In the analysis conducted by Sophie Le Guellec on 160 cases of MPNSTs from the French Sarcoma Group database (36), it was found that MPNST patients who received radiotherapy had higher OS and PFS than those who did not receive radiotherapy. Among patients who underwent surgery followed by radiotherapy, OS and PFS were also significantly higher than in those who received surgery alone. T Valentin (14) and Chengjun Yao (36) also found that the addition of radiotherapy in MPNST patients who underwent surgical resection could improve their PFS and OS rates. In contrast, Bonvalot S (35) observed in a clinical trial of 150 patients with primary retroperitoneal sarcoma (PRRS) that patients who received preoperative radiotherapy plus surgical treatment had higher PFS rates after surgery than those who underwent surgery alone. In our study, only one patient received IORT, and while the prognosis was favorable, the limited number of cases presents obvious constraints. Nevertheless, this offers some insights that IORT may be beneficial for patients with retroperitoneal tumors. In a prospective randomized trial aimed at assessing the efficacy of IORT for retroperitoneal soft tissue sarcoma, it was found that patients who underwent IORT in combination with postoperative radiotherapy experienced reduced local recurrence and radiation-related abdominal complications (37). Additionally, several studies have reported that larger cohorts of patients receiving IORT and External Beam Radiotherapy achieved five-year local control (LC) rates of 50%–70% (38–40). In addition, in our study, only two patients received radiotherapy before surgery, and thus the results may not be representative. Therefore, we believe that the optimal treatment approach for MPNST is surgical resection followed by postoperative radiotherapy. The reason why postoperative radiotherapy can improve the cure rate is that it can kill residual cancer cells after surgery, reduce the risk of cancer recurrence, and help control cancer metastasis, slow down disease progression, and improve patient survival. For tumors that cannot be resected, radiotherapy can reduce the tumor size and decrease its invasion into surrounding tissues.

### High-low risk group

After conducting a previous univariate cox proportional hazards analysis, we found that the use of surgery, tumor metastasis, and radiation therapy had significant effects on the prognosis of patients. Therefore, we grouped the 52 patients in the study into low-risk and high-risk groups based on these three identified risk factors. After grouping, we conducted univariate cox proportional hazards analysis for the high-risk and low-risk groups, resulting the HR was 2.221 (95% CI 0.964–5.121, *p* = 0.061). At the same time, the survival curve plotted using the Kaplan-Meier method revealed a trend of survival difference between the high-risk and low-risk groups. In addition, ROC analysis showed that the AUC of the high-low risk grouping model reached 0.845, indicating good discrimination in predicting the prognosis of RMPNST patients. Therefore, we believe that grouping RMPNST patients into high-risk and low-risk groups has important clinical significance. Firstly, the categorization aids in selecting appropriate treatment strategies. A well-rounded treatment regime that includes surgery followed by radiation therapy has been shown to significantly enhance survival rates among patients. As such, for patients with RMPNST, prioritizing this strategy over chemotherapy is advisable. Secondly, classifying patients enhances the focus on their care. Those placed in the high-risk category should be subject to more intensive monitoring and therapeutic interventions. Lastly, during patient consultations concerning treatment options, recommendations can be tailored based on the risk categorization. For instance, the significance of surgical interventions may be highlighted for those in the low-risk category, whereas a more aggressive pursuit of comprehensive treatment plans is essential for high-risk patients. It is worth noting that for patients in high-risk groups, most non-surgical cases belong to late stage patients, and surgical intervention is not feasible. Therefore, the incidence of tumor metastasis is relatively high, and even radiotherapy is only a palliative measure rather than a cure, leading to inevitable poor prognosis. Our contribution lies in adopting an actionable quantitative evaluation method.

There are several limitations to this work. Firstly, as this is a retrospective study, there may be issues with missing data, recall bias, and initial medical record errors. Due to the lack of specific information on patient comorbidities, genetic predisposition, and factors related to actual surgical resection in the SEER database, these factors were not included in the discussion of this article. Secondly, due to the rarity of RMPNST, patient data is limited, and the *p*-values from the univariate analysis for radiation therapy and high-risk vs. low-risk grouping did not reach statistical significance. However, we believe that they are meaningful because the survival curves are markedly different, and this may be due to the small number of patients. Thirdly, although the performance of the high-risk and low-risk groups is still satisfactory in our cohort, external validation on RMPNST patients from other medical institutions is needed.

## Conclusion

In summary, based on the review and analysis of 52 cases of primary RMPNST from the SEER database, we have identified and confirmed surgery, tumor metastasis, and radiation therapy as significant factors influencing patient prognosis. Additionally, we have successfully established the first high and low-risk stratification model for RMPNST. We believe that comprehensive treatment including surgery and postoperative radiotherapy may be an effective way for RMPNST patients, but a larger sample size is needed to verify.

## Data Availability

Publicly available datasets were analyzed in this study. This data can be found here: https://seer.cancer.gov/data-software/.
